# Supplementary material for: Mechanisms governing the pioneering and redistribution capabilities of the non-classical pioneer PU.1
Source: Nat Commun. 2020 Jan 21;11:402. doi: 10.1038/s41467-019-13960-2 (PMC6972792; doi:10.1038/s41467-019-13960-2)
Supplement: Supplementary file 7 — Source data [file 41467_2019_13960_MOESM7_ESM.zip › Source_Data/FigureS2/FigureS2B_MotifScanOutput/KmeansCluster7/homerResults/motif8.similar.html]

motif8

## Information for motif8

C
T
G
A
A
C
T
G
T
C
A
G
C
T
G
A
T
C
G
A
T
C
G
A
G
T
A
C
  
Reverse Opposite:  

C
A
T
G
A
G
C
T
A
G
C
T
G
A
C
T
A
G
T
C
T
G
A
C
G
A
C
T
  

|  |  |
| --- | --- |
| p-value: | 1e-150 |
| log p-value: | -3.461e+02 |
| Information Content per bp: | 1.668 |
| Number of Target Sequences with motif | 1149.0 |
| Percentage of Target Sequences with motif | 35.79% |
| Number of Background Sequences with motif | 6971.1 |
| Percentage of Background Sequences with motif | 15.94% |
| Average Position of motif in Targets | 102.7 +/- 55.3bp |
| Average Position of motif in Background | 101.1 +/- 74.5bp |
| Strand Bias (log2 ratio + to - strand density) | 0.1 |
| Multiplicity (# of sites on avg that occur together) | 1.10 |
| Motif File: | file (matrix) reverse opposite |

### Similar de novo motifs found

|  |  |  |  |  |  |  |  |
| --- | --- | --- | --- | --- | --- | --- | --- |
| Rank | Match Score | Redundant Motif | P-value | log P-value | % of Targets | % of Background | Motif file |
| 1 | 0.797 | C G T A A C T G A C T G C G T A C G T A A G T C A G T C | 1e-38 | -87.854928 | 8.38% | 3.27% | motif file (matrix) |
| 2 | 0.768 | T C A G A T C G A C T G C G T A C G T A C G T A A C G T | 1e-35 | -82.354487 | 18.38% | 10.61% | motif file (matrix) |
